# Effective utilisation of rapid infusion catheters in perioperative care: a narrative review

**DOI:** 10.1016/j.bjao.2024.100365

**Published:** 2025-01-22

**Authors:** Adam Scorer, Rani Chahal, Louise Ellard, Paul S. Myles, William P.L. Bradley

**Affiliations:** 1 Department of Anaesthesiology and Perioperative Medicine, The Alfred, Melbourne, VIC, Australia; 2 Department of Critical Care, University of Melbourne, VIC, Australia; 3 Department of Anaesthesia, Perioperative and Pain Medicine, The Peter MacCallum Cancer Centre, Melbourne, VIC, Australia; 4 Department of Anaesthesia, Austin Health, Melbourne, VIC, Australia; 5 Safe Airway Society, Australia & New Zealand, Australia; 6 School of Translational Medicine, Faculty of Medicine, Nursing and Health Sciences, Monash University, Melbourne, VIC, Australia; 7 Anaesthetic Advisory Committee, Epworth Healthcare, Melbourne, VIC, Australia; 8 Anaesthetic Subcommittee, Victorian Perioperative Consultative Council, Safer Care Victoria, Melbourne, VIC, Australia

**Keywords:** cannula, catheter, equipment and supplies, intravenous infusions, peripheral venous catheterisation, vascular access devices

## Abstract

The Rapid Infusion Catheter (RIC) has transformed intravenous (i.v.) access, allowing for rapid fluid delivery peripherally. It may negate the need for a central vein sheath to be placed. This review explores the clinical utility of RICs while addressing technical considerations and potential risks.

The RIC is a large-bore i. v. sheath available in two sizes. Its maximal flow rate is 1200 ml min^−1^, making it advantageous in significant blood loss scenarios such as trauma and major surgeries. Insertion involves the Seldinger technique.

Monitoring and maintaining the RIC is crucial to detect and address immediate complications such as occlusions, infiltration, phlebitis, and extravasation of infusate. Although the related complications share similarities with those of other peripheral i. v. cannulae, they have a lower risk of occlusion and accidental displacement. Catheter removal should be considered once the patient is stable or alternative access is available to avoid infectious complications. Removal of the RIC needs to be performed by those educated in RIC management.

Maximal flow rate is an essential factor in assessing the performance of i. v. cannulae, and studies have shown that RICs outperform other peripheral and central catheters in this regard.

In conclusion, RIC offers advantages over large-bore central venous access for large-volume rapid infusions, including ease of insertion and reduced severe complications. The RIC demonstrates lower thrombosis rates and a different complication profile than peripherally inserted central catheters.

Understanding the characteristics and applications of RICs can help healthcare professionals make informed decisions about their use in various medical scenarios.

Peripheral i. v. cannulation is a critical procedure that provides access for administering fluids, medications, and blood products. It is essential for the safe perioperative management of both elective and emergent surgical patients. The choice of vascular access in these scenarios requires careful consideration, weighing the risks and benefits specific to each patient and procedure. Particularly for patients at risk of massive transfusion, such as those experiencing major trauma, undergoing complex surgery, or facing significant blood loss, effective fluid resuscitation and oxygen delivery are paramount.^1^ In these cases, large-bore peripheral or central venous access is necessary to support rapid fluid administration and maintain hemodynamic stability.

Large-bore peripheral intravenous cannulas (PIVCs) are devices designed for these high-demand situations and can be lifesaving. The American College of Surgeons' Advanced Trauma Life Support (ATLS) guidelines^2^ define a large-bore PIVC as an i. v. catheter with a gauge size of 18 Ga or larger, with lower numbers indicating a larger internal diameter. These cannulas allow for the rapid administration of fluids, blood products, and medications, making them a crucial tool in trauma resuscitation, major surgery, and in patients requiring high-volume transfusion.

However, not all large-bore PIVCs are created equal—some achieve substantially faster flow rates than others. One notable PIVC example of this is the Rapid Infusion Catheter (RIC), developed by Arrow® International (Teleflex, Morrisville, NC, USA). The RIC is specifically designed to deliver fluids and blood products at extremely high flow rates, up to 1200 ml min^−1^.^3^

The RIC comes in two sizes: a 5-cm 7-Fr sheath, roughly equivalent to 13.3 Ga, and a 6.4-cm 8.5-Fr sheath,^4^ approximately equivalent to 11.8 Ga. These sheaths allow rapid access without the need for central venous catheter placement, offering a quicker and safer option in emergencies.

The utility of RIC lines extends across multiple domains, including the prehospital setting, trauma (both civilian and military), open aortic surgery, major spine surgery, liver surgery, cancer surgery, and cases involving placental abnormalities or at risk of significant haemorrhage.^5^, ^6^, ^7^, ^8^, ^9^, ^10^, ^11^ The RIC provides a practical solution for patients requiring wide-bore access, reducing the risks associated with central line placement, such as pneumothorax or vascular injury. Furthermore, using RICs can free up central access for other critical functions, such as continuous venovenous hemofiltration, a pulmonary artery catheter insertion, or the administration of multiple vasoactive agents. Despite their potential advantages, the widespread adoption of RIC lines is limited by a lack of appreciation of their use and limited robust clinical data on their specific benefits and possible complications.

Clinical indications for using RICs are not explicitly described in existing guidelines, and the paucity of comparative studies with other forms of vascular access restricts their routine use in many institutions. Additionally, concerns about complications, such as extravasation, phlebitis, and other insertion-related issues, require further investigation.

In recent post-COVID developments, one of the authors identified a significant gap in staff understanding of RICs when issues were raised concerning their management and removal in the postoperative period. This may partly be explained by the reduced surgeries performed during the pandemic, which led to less exposure and experience using RICs, as the issues raised had not been a concern earlier.

Further confusion arose when the staff sought clarity from a company representative who classified RICs as i. v. sheaths but not a PIVC and recommended alternatives to be used such as central venous access devices (CVADs), other large-bore PIVCs, peripherally inserted central catheter (PICCs), or an intraosseous needle (*email communication 12 August 2022*). This classification and recommendation overlook the clinical reasons for selecting RICs and ignore the alternative devices' differing risk profiles and flow characteristics. This was further compounded at the hospital level by misinterpreting the RIC size, with the 8-Fr sheath mistakenly being considered an 8-Ga catheter.

This misinterpretation probably occurred because two different measurement systems can be used for i. v. devices: the Birmingham wire gauge system (used for needles and cannulas) and the Charrière system (used for catheters), also known as the French gauge or French (Fr).

Both systems measure the devices' outer diameter but function differently. The Birmingham system operates inversely, meaning a smaller gauge number corresponds to a larger diameter. In contrast, the French gauge system follows a direct metric approach, where 1 French equals one-third of a millimetre, and the size increases as the French number increases. This difference in how the systems assign size can easily lead to confusion when comparing or interpreting device sizes, as with misinterpreting the 8-Fr sheath as an 8-Ga catheter.

This narrative review aims to provide an evidence-based summary of the indications, benefits, and potential complications of RIC lines, particularly in the context of trauma, resuscitation, and elective major surgeries. The review also includes a guide to safely inserting, maintaining, and removing these devices, thereby facilitating their incorporation into clinical practice guidelines. By exploring the merits of RIC lines and comparing them with both peripheral and central access alternatives, this review seeks to establish a more transparent framework for their appropriate use and facilitate easy adoption within clinical guidelines.

## Methods

This narrative review was conducted following the principles outlined in the Preferred Reporting Items for Systematic Reviews and Meta-Analyses (PRISMA).^12^

Ethical approval was not sought for this study as it consisted solely of a literature review involving the analysis of existing research without collecting new or individual personal data from human or animal subjects.

A literature search was conducted to find relevant studies, clinical guidelines, case reports, and expert opinions on RICs and their associated morbidities. The search utilised databases PubMed, Cochrane Library, Google Scholar, and grey literature sources, including ClinicalTrials.gov and Google utilising various search terms, keywords, and combinations using Boolean operators. These search terms included ‘Rapid Infusion Catheter’, ‘peripheral inserted large-bore intravenous cannulas’, ‘large bore’, ‘intravenous cannulae (and variations PIVC, PIV)’, ‘rapid fluid administration’, ‘trauma’, ‘resuscitation’, and ‘elective major surgeries’.

Moreover, additional relevant articles were retrieved from the references of key publications. No prospective RCTs were identified. Each document from the identified articles was carefully screened, including case series, case studies, and opinion articles. In the review process subsequent articles were suggested and reviewed for relevance.

It encompassed all articles published up to December 2023 and did not impose any language restrictions. The inclusion criteria included any mention of RICs.

The articles were imported into Covidence workflow platform software (Melbourne, VIC, Australia) for screening and assessing for eligibility.

### Papers identified

**Fig. 1:**
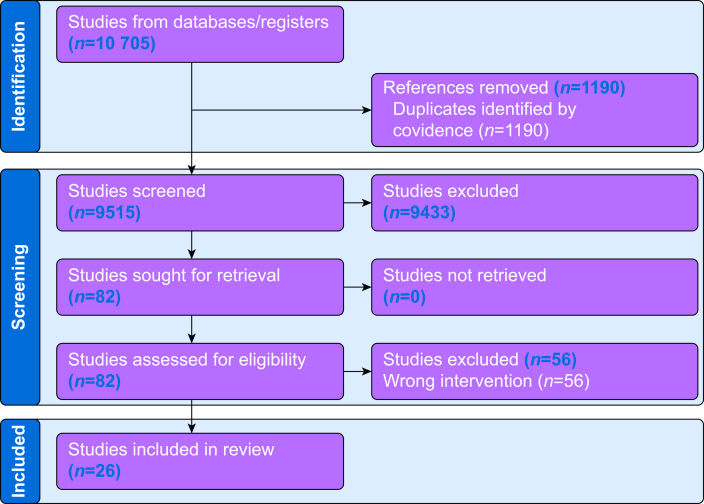
Flow Chart of Preferred Reporting Items for Systematic Reviews and Meta-Analyses (PRISMA) for the comprehensive narrative review of Rapid Infusion Catheters in perioperative care.

A total of 10 705 references were identified and entered into Covidence™ for screening. After identifying 1190 duplicates, 9515 studies remained for title and abstract screening. Of these, 9433 were excluded based on their titles and abstracts. Subsequently, 82 studies were assessed for full-text eligibility, and 56 were excluded for involving the wrong intervention. Ultimately, 26 articles or studies met the inclusion criteria and were included in the review (Fig 1 ).

### Flow rate

Hagen–Poiseuille's formula describes laminar flow of an incompressible, Newtonian fluid in a rigid tube with circular cross-section as: $Q=\frac{\pi \;r^{4}\Delta P}{8\eta L}$

Where *Q* is the flow rate, *r* is radius, Δ*P* is the pressure difference between the two ends of the tube, *η* is dynamic viscosity, and *L* is the length of the tube.

Laminar flow is proportional to the radius of the fourth power. It is the most important determinant of laminar flow. If all other factors remain constant, the flow rate increases 16 times for each radius doubling. Thus, an 8.5-Fr, 64-mm RIC will have an 11 times greater flow rate than an 18-Ga, 33-mm peripheral cannula under laminar flow conditions. Under physiological conditions, where blood is a non-Newtonian fluid, vessels are not uniform rigid tubes, flow is often turbulent, and formula accuracy is degraded. However, the relative contribution of the determinants remains unchanged. The manufacturer-quoted flow rates are 572 ml min^−1^ for the 8.5-Fr RIC and 500 ml min^−1^ for the 7-Fr RIC.^13^ Several studies have examined the flow rates of this and other devices under simulated clinical conditions (Table 1).

**Table 1:** Maximum infusion rates for peripheral and central vascular access devices.

Authors				Manufacturers' flow rates (Braun)	Wrenn and colleagues, 2017 (Boston Children's hospital	Emergency trauma Manual 2015	Brown and colleagues, 2008	Barcelona and colleagues, 2003	Khoyratty and colleagues, 2016	Milne and colleagues, 2021
Devices	Number of lumens	Outer diameter (mm)	Lumen gauge size	(ml min^−1^)	Maximum flow rates (ml min^−1^)	100-Ml infusion time via rapid infuser (s)	Flow rate through level 1 Warmer (ml min^−1^)	Flow rate through level 1 Warmer (ml min^−1^)	Mean flow rate with standard giving set (ml min^−1^)	Mean maximum flow with RBC and FFP (ml min^−1^)
PIVC	1	0.7	24	22 (19 mm length)	17					
PIVC	1	0.9	22	35 (25 mm length)	35				36	
PIVC	1	1.1	20	60 (32 mm length)	60	407	138.7	140	66	
PIVC	1	1.3	18	105 (32 mm length)	105	263	212.1	209	90	183
PIVC	1	1.7	16	215 (32 mm length)	220	140	391.2	368	156	448
PIVC	1	2.2	14	350 (32 mm length)	330	90	483.7	488	198	728
Arrow Rapid infusion Catheter (RIC) 7 Fr	1	2.3	13.3	500 (51 mm length)	1137	60	603.3	564		1000
Arrow RIC 8.5 Fr	1	2.8	11.8	572 (64 mm length)	1200	46		596	222	
										
PICC 4 Fr	1		18	17.2	21.2			450		
PICC 5 Fr	1		18		19.8			533		
PICC 5 Fr	2		18/18	9.5 (18 Ga proximal) 9.5 (18 Ga distal)	9.6					
PICC 6 Fr	2		18/18		12.6			548		
PICC 5 Fr	3		18/20/20		16.3/2.2					
PICC 6 Fr	3		18/19/19	19.0 (18 Ga) 5.4 (19 Ga medial) 5.6 (19 Ga distal)	19.4/4.6					
MAC Introducer 12-Ga lumen: 9 FG	2		12	524 (9 FG) 149 (12 Ga)	337					1000
MAC Introducer 11.2-Ga lumen: 9 FG	2		11.2		1200					
Central sheath introducer 8.5 FG	1			500	1156	65	594.6			
Central sheath introducer 6 FG	1					130				
Angiocath 14 Ga (13.3 cm)	1					130				
14-Ga four-lumen central line	4		14 16 18 18	71.9 (14 Ga) 38.1 (16 Ga) 19.4 (18 Ga proximal) 18.0 (18 Ga medial)		320			84	

#### Standard blood/solution pump set

Khoyratty and colleagues^14^ found that a latex-free, 200-cm set length with priming volume 70 ml (Alaris Products, Ashford, UK) hung 100 cm above the i.v. device infuses Plasma-Lyte 2.5 times faster with an 8.5-Fr RIC (222 ml min^−1^) than an 18-Ga cannula (90 ml min^−1^). Adding a needle-free valve to the system significantly decreases flow rates in 16-Ga devices or larger. This flow rate reduction is exaggerated with increasing internal diameter of the catheter (36% reduction with 14-Ga cannula compared with a 47% reduction with 8.5-Fr RIC). Adding a pressure bag set to 300 mm Hg effectively doubles the flow rate of the 8.5-Fr RIC from approximately 200 ml min^−1^ to 500 ml min^−1^, provided no needle-free valves are used.^14^ When they compared published data by Barcelona and colleagues,^15^ the authors concluded that smaller diameter cannulas using a rapid infusion system did not significantly improve flow rates over a pressure bag system. Better flow rates were only achieved with 7-Fr to 8.5-Fr large-volume infusion catheters.^15^ Milne and colleagues^6^ performed an *in vitro* study that examined infusing a mixture of red blood cells and fresh frozen plasma in a 1:1 ratio going through a rapid infusion device and found that 7 Fr had a flow rate of 1000 ml min^−1^ with or without a patient line extension.

#### Level 1 warmer (rapid fluid delivery system)

Brown and colleagues^16^ found that the 7-Fr RIC has the fastest infusion rate when combined with a Level 1 Warmer. It is marginally faster than the 8.5-Fr central sheath introducer (603 *vs* 601 ml min^−1^) and significantly quicker than 14-Ga (30 mm 483 ml min^−1^), 16-Ga (391 ml min^−1^), and 18-Ga (212 ml min^−1^) peripheral cannulas. Barcelona and colleagues^15^ identified similar flow rates with the 8.5-Fr central sheath introducer of 596 ml min^−1^ with the Level 1 Warmer and rates not dissimilar with the 14-Ga (488 ml min^−1^), 16-Ga (368 ml min^−1^), and 18-Ga (209 ml min^−1^). The superior flow rates achievable with RICs are reinforced by a practical assessment run by an Emergency Trauma Management Course.^17^ They demonstrated that the 8.5-Fr RIC can infuse 1000 ml of crystalloid in 45 s, followed by the 7-Fr RIC (60 s), 8.5-Fr central sheath introducer (65 s), 14-Ga (90 s), 16-Ga (140 s), 18-Ga (263 s), and 14-Ga four-lumen central venous catheter (320 s).^17^

When used with a Rapid Infusion System, the 8.5-Fr RIC outperformed all tested central and peripheral i. v. devices, with flow rates up to 1200 ml min^−1^ for normal saline and Hetastarch. This includes the 9-Fr multi-lumen access catheter (MAC) introducer (Teleflex, Morrisville, NC, USA), which could also achieve flow rates of 1200 ml min^−1^ with normal saline but only 1130 ml min^−1^ with Hetastarch. The 8.5-Fr RIC consistently provides the fastest flow rates across various conditions, including fluid viscosity and delivery modality.^14^^,^^16^, ^17^, ^18^ These devices may best serve patients requiring access for rapid fluid resuscitation.

### Clinical indications

#### Haemorrhagic shock: trauma

Major haemorrhage is the leading cause of shock in the trauma patient. It is the first and second most common cause of death in military and civilian trauma, respectively.^19^^,^^20^ More importantly, it represents the leading cause of preventable death in both settings.^20^^,^^21^ The median time from the onset of haemorrhagic shock and death is 2 h.^22^ Early intervention in the prehospital and hospital settings is vital.

Early intervention comprises haemorrhage control and i.v. fluid resuscitation. These interventions maintain intravascular volume and oxygen-carrying capacity, protect endothelium, and prevent dilutional and traumatic coagulopathy.^23^ These interventions prevent early death related to severe haemorrhage and improve outcomes in those surviving surgery and critical care by preventing organ failure as a result of tissue ischaemia.^21^

RICs are well suited to providing early, rapid, and balanced resuscitation. They are quick and easy to insert and can be used to upgrade existing peripheral access and avoid complications associated with central venous access.^7^^,^^24^^,^^25^ RICs have a three-fold reduction in complication rates (∼1.6%) compared with central venous catheterisation (4.7%).^25^ Access to the patient is often limited by simultaneous assessment and management of immediate life threats, including airway obstruction and severe chest injuries. The utility of a large-bore peripheral device removes conflict for therapeutic space in the neck and chest required for central access. They can be inserted by non-physician prehospital practitioners and junior medical staff,^25^ allowing senior medical staff to focus on more advanced interventions and decision-making.

#### Abdominal aortic surgery

Abdominal aortic surgery is conducted by open or endovascular approaches, in either an elective or emergent setting.^26^ Morbidity and mortality of abdominal aortic surgery is high, often resulting in cardiovascular, respiratory, and renal complications.^26^^,^^27^ Mortality ranges from 2–5% in elective endovascular and open repairs, to >50% in ruptured abdominal aortic aneurysm.^27^

Blood loss is variable and increases with open *vs* endovascular and emergent *vs* elective procedures.^28^ Haemorrhage owing to rupture is the major cause of death in non-surgically treated patients.^27^ There is potential for significant fluid shift, blood loss, and labile haemodynamics with aortic clamping and unclamping (open procedures). Despite these conditions, anaesthesiologists must minimise haemodynamic changes to reduce aortic wall stress and optimise surgical conditions. To do so, they need reliable large-bore and central venous access for rapid volume replacement and vasoactive medications to maintain intravascular volume and oxygen delivery to all vascular beds, preventing or reducing perioperative complications.^27^^,^^29^^,^^30^

RICs are well suited for use in abdominal aortic surgery, with greatest utility in open and emergent procedures. In elective endovascular cases, large-bore peripheral venous catheter can be quickly accessed and upgraded in rare cases of a rupture or major bleeding. In emergent endovascular and open cases, they are faster and easier to insert,^25^ allowing early lifesaving volume replacement. In elective open abdominal aortic surgery, they provide a peripheral means of rapid volume replacement in case of rupture or major bleeding. This also allows the central access device to be used solely for vasoactive medications to maintain tight haemodynamic control. This is crucial in reducing aortic wall stress (precipitating rupture) and smoothing the haemodynamic effects of aortic clamping and unclamping. Dedicated central access for these agents allows more lumens for different agents and avoids bolusing these drugs with concomitant volume infusion.

Risk of major bleeding persists into the postoperative phase of care.^28^ In open procedures, aortic graft anastomosis disruption may occur in the early postoperative phase and requires tight haemodynamic control. Endovascular procedures are at a lower risk, but bleeding can be significant, occurring at access sites or anywhere along the aorta or iliofemoral arteries.^28^ When used, large-bore i.v. access, including RIC, should remain *in situ* until the risk of bleeding is deemed acceptably low. Removing any large-bore i.v. device should be individualised and physician-led.

#### Major spine surgery

The risk of bleeding with major spinal surgery can be high, resulting in significant patient morbidity.^31^ Risk factors for blood loss include advancing age, increasing number of levels instrumented, tumour surgery, increased abdominal pressure, and osteotomy.^32^, ^33^, ^34^, ^35^ Average blood loss ranges from 800 ml in non-instrumented fusions to 1500 ml in instrumented fusions, up to 7000 ml.^36^ The consequences of such losses in major spinal surgery include haemodynamic instability, reduced tissue oxygen delivery, and increased venous thromboembolism. Those undergoing multilevel fusions with major blood loss are at increased risk of visual loss owing to ischaemic optic neuropathy.^37^ The risk of bleeding continues into the postoperative period, with approximately 15% of patients requiring transfusions in the ensuing 24 h.^32^

RICs are ideally suited to spinal surgery to keep up with these potentially enormous losses. Patients are often in prone positions where access to upgrade or place central venous catheters is impossible. These devices are best placed before the commencement of surgery. Given the risk of ongoing bleeding in the postoperative period, these devices should remain *in situ* until the surgical or perioperative team deems the bleeding risk acceptable.

#### Cancer surgery

Excessive bleeding is common in cancer surgery as a result of local (invasion, angiogenesis) and systemic effects of cancer (tumour-related fibrinolysis), anticancer therapies, or thromboembolism prophylaxis. Blood loss is also an inherent risk of major solid malignancy surgery. These compounding factors place patients at increased risk for major intraoperative bleeding.

With newer and more aggressive surgical interventions being used to improve oncological outcomes, massive intraoperative and postoperative haemorrhage and blood transfusions are not uncommon, especially in the setting of major debulking surgery. This may be anticipated or unexpected. Tumour location, vascularity, and proximity or invasion of major vessels are key considerations when planning for massive blood loss.

The rate of perioperative red cell transfusion ranges from 9.4% to 90%, depending on the type of surgery and patient factors.^38^, ^39^, ^40^ However, the exact incidence of massive transfusions is unknown. Procedures associated with perioperative transfusion of greater than 10 units of packed red cells include extrapleural pneumonectomies, radical nephrectomy with inferior vena cava (IVC) thrombectomy, total pelvic exenterations, liver resections, hemipelvectomy, spine, and sacral surgery.^41^ Massive blood loss in sacral resections is almost universal, with reported losses of up to 37 L.^42^^,^^43^

The above procedures would benefit from inserting the RIC as blood loss can be torrential and sudden, requiring a rapid infusion of blood product to restore blood volume and haemostasis. Although the incidence of use of RIC with these surgeries is unknown, inserting a peripheral venous catheter is beneficial in cancer patients as they present with implantable chemotherapy ports that can complicate the insertion of central large-bore venous catheters. However, it is imperative that visualisation of the site of RIC access is possible to ensure patency of the catheter and exclude extravasation of blood products, especially when rapid infusion systems are used.

#### Liver surgery

It is routine practice in many liver transplant centres to use unilateral or bilateral RIC cannulas for volume resuscitation during liver transplant,^11^ 2×8.5-Fr RIC cannulas are inserted, usually one in each arm, in addition to central venous access with a 9-FG central sheath and pulmonary artery catheter.

A rapid infusion system (fluid management system [FMS]) is connected via a ‘Y’ connector to these two RICs and is used to provide fluid resuscitation. In a liver transplant, although the average fluid transfusion is approximately 12 L, it is not uncommon for transfusion volumes to exceed 20 L, with recorded transfusion volumes of >100 L at times (Reference: *Data were prospectively collected from the FileMaker Database - Victorian Liver & Intestinal Transplant Unit, Melbourne, Australia).* This transfusion requirement is generally concentrated around the hepatectomy phase of the transplant, necessitating large-volume transfusion over a short period. Using the FMS, maximum delivery rates can be set to 750 ml min^−1^, and a second system can be utilised if required. It is essential that the i. v. access can facilitate these rates of transfusion.

For complex non-transplant liver surgery, including extended right hemi-hepatectomy procedures involving resections close to the IVC, resections in patients with early or moderate cirrhosis, and those with significant portal hypertension, RIC is sometimes utilised. For the small subset of patients who require the use of venovenous bypass for liver resection surgery (because of surgical complexity and need for an extended period of IVC, portal venous clamping, or both and thus cold perfusion of the liver), bleeding after weaning from bypass and resection is common. In these patients, RIC allows for rapid, reliable infusion of large volumes of blood and other fluids.

Using RIC is useful for IVC tumour resections, especially in stage 3 or 4 tumours, where cardiopulmonary bypass, systemic hypothermia, and heparinisation is used. Bleeding is especially problematic in such patients, mandating the ability to give fluid rapidly.

#### Placenta accreta spectrum disorders

Placenta accreta is when the placenta invades the uterine wall and becomes inseparable. Placenta accreta spectrum (PAS) is the term used to describe the three subtypes with increasing invasion: placenta accreta vera, placenta increta, and placenta percreta.^44^ In its most severe form (percreta), it can invade surrounding organs, including the bladder and bowel.^44^ PAS can be identified antenatally with subsequent multidisciplinary management in a tertiary obstetric facility. However, it can be encountered unexpectedly with subsequent increased risk of complications, including catastrophic blood loss.^45^, ^46^, ^47^, ^48^

PAS creates a hypervascular pelvis with an increased number of pathological vessels prone to bleeding.^49^ The placenta does not separate properly at delivery, resulting in ongoing atony and bleeding. However, the use of oxytocics can make bleeding worse by encouraging the separation of the invasive placenta.^49^ Their use is not routine and is reserved for uterus-sparing procedures with ongoing bleeding as a result of atony or where the placenta has separated.^45^ Resection of placental tissue from the uterus and surrounding tissue can also result in significant blood loss.^44^^,^^45^^,^^49^ Unsurprisingly, PAS is a significant cause of major obstetric haemorrhage and severe maternal morbidity in the developed world.^49^

Blood loss ranges from around 2 L in the elective setting to 8 L or more in the emergent setting.^47^^,^^50^ Most blood loss occurs post-delivery but may occur pre-delivery in the emergent setting.^46^ It can be rapid and torrential, and all guidelines for the management of PAS advocate bilateral large-bore peripheral i.v. access. Few advocate for the use of a central venous catheter as the primary problem is hypovolaemia, which does not require centrally infused vasoactive medications. Central access is reserved for those with difficult large-bore peripheral access or patients with other comorbidities requiring central access (e.g. a mother with concomitant cardiac disease).^44^^,^^45^^,^^49^^,^^50^ RICs are ideally suited in elective and emergent settings owing to ease of insertion, increased patient comfort, and ability to place bilateral devices to keep up with such rapid losses.^49^ They can also upgrade existing i.v. access for unexpected PAS and significant bleeding.^51^

### Rapid Infusion Catheter Insertion technique

To ensure appropriate operator knowledge and skill in using RIC, familiarisation with the manufacturer's insertion information package^4^ and device training should be undertaken. The placement of RIC involves a Seldinger technique.

It is important to identify whether the patient has the appropriate indications for inserting the sheath,^52^ and consent for this procedure may be required.

An appropriate vein needs to be selected, and its location and size assessed. An ultrasound can help reduce the potential for insertion-related complications^53^, ^54^, ^55^ by identifying the vein's diameter, path, and patency, along with other structures, such as arteries or nerves, that might be nearby.

Ideally, for an optimal catheter-to-vessel ratio (1:3 or less), the vein's diameter should be three times the catheter's diameter or approximately 7–9 mm for an 8.5-Fr RIC, where 1 Fr is equal to one-third of a millimetre in diameter (i.e. a 9 Fr has a 3-mm [9/3] diameter), to minimise the risk of vessel trauma and thrombosis and increase the insertion success rate.^55^^,^^56^ The path should ideally be straight and of a length >6 cm, so it accommodates at least 2.75 cm or more of RIC to prolong the median survival time of the catheter.^57^ Typical sites that might be chosen are the median cubital, basilic or cephalic veins of the arm, less commonly the saphenous, or rarely external jugular vein,^58^ depending on the location of the surgery or injury. These catheters are intended for short-term volume replacement, and although it is preferable to avoid the antecubital fossa owing to its location in an area of flexion, it may still be used when necessary.

The steps for the insertion include: (1) Identify, prepare, and clean the insertion site with chlorhexidine and adhere to aseptic technique. (2) Local anaesthesia is performed if the patient is conscious. (3) Insert a 20-Ga cannula into the target vein and confirm intravascular placement, or alternatively, place a long 18-Ga cannula, which has the added advantage of ensuring an appropriate straight length of vein if ultrasound is unavailable. (4) Introduce the guidewire. If resistance is encountered, try simple manipulation attempts to get past a valve or reinsertion once to reconfirm intravascular placement. (5) Once the guidewire is in place, remove the 20-Ga cannula while always maintaining a grip on the wire. It is helpful to have some sterile gauze in case of bleeding. The proximal loss of the guidewire into a central vein should be a ‘never event’. (6) If necessary, use the supplied scalpel to make an incision in the skin with the sharp edge away from the guidewire to avoid cutting it. (7) Advance the dilator and sheath over the guidewire with a slight twisting motion. Avoid excessive force to prevent damage to the sheath or trauma to surrounding structures, such as arteries or nearby nerves. Ensure the guidewire, dilator, and sheath are aligned, and confirm that the guidewire is not lost within the vein at any point. (8) Advance the sheath of the dilator. (9) Remove the dilator and wire while holding the sheath in the vein. (10) Secure the sheath with a dressing and connect to the Luer-lock i.v. fluid line or needle-free connector (note that the needle-free connector might reduce the flow rate). A suture may be used to secure the catheter, but care should be taken to avoid damage or ligation of the sheath. (11) Apply a sterile, transparent, semipermeable adhesive dressing. A chlorhexidine-impregnated dressing commonly used for central venous catheters is a good choice. (12) Finally, confirm proper placement of the cannula with a 20-ml bolus of i. v. fluid to check the flow, and no infiltration or extravasation.

It must be highlighted that the dilator should be removed before attaching the i. v. fluid line to avoid potentially serious complications,^59^ especially when using a high-pressure rapid infusion system.

### Monitoring and maintenance

Nurses play a vital role in diligently monitoring i.v. catheters, and their prompt intervention can minimise complications and ensure patient safety. It has been recommended that i. v. catheters be assessed at least once a shift and more frequently in acutely ill patients^60^^,^^61^ for signs of complications, including occlusions, infection, damaged catheter, extravasation, phlebitis, infiltration, and thrombosis. A visual infusion phlebitis score or a clinical decision-making tool^62^ can help guide the catheter's ongoing management. There should be a regular review of the continuing medical need for the catheter so that early removal can take place to minimise the infection risk from i.v. lines. In practical terms, this is usually after the patient has been haemodynamically stable for 12–24 h and unlikely to require any further rapid large-volume infusion. An alternate, smaller i.v. access device should be placed at this stage.

### Rapid Infusion Catheter removal

The removal of RIC is not dissimilar to that of a smaller gauge cannula; however, a stitch cutter is required (if it has been sutured in) and the application of sterile gauze with firm pressure on the site for 5 min or until the bleeding has resolved. Once the bleeding has stopped, the gauze can be removed, the site cleaned, and a sterile adhesive dressing applied. Signs of bleeding should be monitored as there is a slight chance that the entry site may require surgical closure with a suture.^10^

### Complications

The complications associated with RIC are like those of any other i.v. catheter inserted in a peripheral vein. They can arise from various aspects of the insertion procedure, therapy duration, or catheter removal.

Regarding RICs, larger cannulae have a lower risk of occlusion and accidental removal or dislodgement than smaller-gauge cannulae.^63^ However, their use carries an increased risk of phlebitis. Phlebitis occurs in approximately 4.6–15.4% of all PIVCs, with a higher incidence in females. Some studies have suggested that using the Seldinger insertion technique may reduce the risk of phlebitis.^64^

Vessel wall perforation during insertion can result in either infiltration or extravasation. Infiltration occurs when non-vesicant solutions leak from the vein into the surrounding tissue, whereas extravasation involves tissue damage owing to the leakage of a vesicant solution. Larger-diameter cannulas, particularly if they damage the posterior wall, may increase the likelihood of these complications.

In a case series involving 839 patients undergoing liver transplantation, the reported rate of RIC extravasation was approximately 1.7%, with no evidence of permanent harm. The odds of extravasation were higher in patients with coagulopathy, indicated by an international normalised ratio >1.4.^10^ In comparison, the mean infiltration rate for PIVC in randomised controlled studies conducted between 1990 and 2014 was 23.9%, significantly higher than that of RICs.^64^

There is one case report of temporal-related skin necrosis associated with a correctly placed RIC insertion in a polytrauma patient undergoing CPR for pulseless electrical activity. However, this case was confounded by fractures in the area and an arterial catheter, making it difficult to attribute the issue solely to RIC. Other perfusion issues were considered more likely culprits.^65^

Subjectively, from one of the author's experiences placing RICs in one to two selected cases per month over 25 yr, there have been few complications. One case involved difficulty in insertion, another involved identified vessel wall perforation with a test bolus of 20 ml of NaCl 0.9%, and another experienced grade 2 mechanical phlebitis. These complications equate to a rate of approximately 1%. The low complication rate may be attributed to RIC being removed early,^66^ usually within 24 h of surgery, when the patient is stable, and the RIC is no longer necessary.

The comparison of complications with other venous access devices shows that RIC has several advantages over large-bore central venous access. It offers a quicker and easier insertion procedure while avoiding severe complications such as pneumothorax, pneumomediastinum, recurrent laryngeal nerve injury, air embolism, and carotid or subclavian artery puncture.^67^ Additionally, RIC does not require specific removal techniques to minimise the risk of venous air embolism. Compared with PICCs, RIC has a lower rate of thrombosis^68^ and a different complication insertion profile, which avoids the potential risks of cardiac tamponade and arrhythmias.^67^

Overall, the flow rate for RICs perform better than all other tested venous access devices and are easy to insert, with a safer insertion profile than comparable large-bore central venous devices whose flow rates are otherwise of a similar magnitude.

### Limitations

#### Weaknesses and strengths of the review

A key limitation of this narrative review is the potential lack of generalisability because of the varied patient populations included in the selected studies. Additionally, the absence of prospective RCTs limits the strength of evidence. This review did not encompass all available databases, so some relevant publications on RICs might have been missed, and a healthcare librarian was not used to locate or streamline the literature searches. Despite these limitations, the strength of this review lies in its analysis of both published literature and grey sources, providing a thorough exploration of RICs in diverse clinical settings. This article addresses the knowledge gaps in the field, particularly where the practical use of RICs has been underexplored and consolidates this information into this review.

#### Future studies

Future research should focus on conducting RCTs to assess the efficacy and safety of RICs in various clinical settings. Studies investigating the education and training of healthcare staff in the proper management of these devices would also be beneficial in addressing the limitations identified in this review.

## Conclusions

The RIC emerges as a transformative tool in i. v. therapy, providing a high-flow, peripheral option that mitigates the need for more invasive central venous access. This innovation is particularly valuable in emergency and operative situations where rapid volume resuscitation is crucial, such as in trauma or massive blood loss during surgery. The RIC's maximum flow rate of 1200 ml min^−1^ coupled with the straightforward Seldinger insertion technique enhances its practicality and safety. Moreover, it offers flexibility with two different sizes to cater to various clinical needs. As a device that potentially lowers the risk of severe complications commonly associated with central venous catheters, RIC's role in acute care settings is both pivotal and promising, demanding greater uptake into clinical guidelines and practice.

## Authors’ contributions

Study conception: PB

Study design: AS, PB

Data acquisition: AS, RS, LE, PB

Data analysis: AS, RS, LE, PM, PB

Data interpretation: AS, RS, LE, PM, PB

Drafting the article: AS, RS, LE, PM, PB

Critical revision of the manuscript for important intellectual content: AS, RS, LE, PM, PB

Approving the final version to be published as well as agreed to be accountable for all aspects of the work, thereby ensuring that questions related to the accuracy or integrity of any part of the work are appropriately investigated and resolved: AS, RS, LE, PM, PB

## Declaration of generative AI in scientific writing

During the preparation of this work, the author(s) used ChatGPT to improve readability and language. After using this tool/service, the author(s) reviewed and edited the content as needed and take(s) full responsibility for the publication's content.

## Declaration of interests

PM is an editor for *BJA Open*. The other authors declare no conflicts of interest.
